# Pharmacological Inhibition of Nicotinamide Phosphoribosyltransferase/Visfatin Enzymatic Activity Identifies a New Inflammatory Pathway Linked to NAD

**DOI:** 10.1371/journal.pone.0002267

**Published:** 2008-05-21

**Authors:** Nathalie Busso, Mahir Karababa, Massimo Nobile, Aline Rolaz, Frédéric Van Gool, Mara Galli, Oberdan Leo, Alexander So, Thibaut De Smedt

**Affiliations:** 1 Service of Rheumatology, Department of Medicine, Centre Hospitalier Universitaire Vaudois and University of Lausanne, Lausanne, Switzerland; 2 TopoTarget Switzerland SA, Lausanne, Switzerland; 3 Laboratoire de Physiologie Animale, Université Libre de Bruxelles, Gosselies, Belgium; Centre de Recherche Public-Santé, Luxembourg

## Abstract

Nicotinamide phosphoribosyltransferase (NAMPT), also known as visfatin, is the rate-limiting enzyme in the salvage pathway of NAD biosynthesis from nicotinamide. Since its expression is upregulated during inflammation, NAMPT represents a novel clinical biomarker in acute lung injury, rheumatoid arthritis, and Crohn's disease. However, its role in disease progression remains unknown. We report here that NAMPT is a key player in inflammatory arthritis. Increased expression of NAMPT was confirmed in mice with collagen-induced arthritis, both in serum and in the arthritic paw. Importantly, a specific competitive inhibitor of NAMPT effectively reduced arthritis severity with comparable activity to etanercept, and decreased pro-inflammatory cytokine secretion in affected joints. Moreover, NAMPT inhibition reduced intracellular NAD concentration in inflammatory cells and circulating TNFα levels during endotoxemia in mice. *In vitro* pharmacological inhibition of NAMPT reduced the intracellular concentration of NAD and pro-inflammatory cytokine secretion by inflammatory cells. Thus, NAMPT links NAD metabolism to inflammatory cytokine secretion by leukocytes, and its inhibition might therefore have therapeutic efficacy in immune-mediated inflammatory disorders.

## Introduction

In humans, chronic inflammatory diseases represent a major medical challenge, both in terms of our understanding of their underlying mechanisms as well as their treatments. In a disease such as rheumatoid arthritis (RA), the pathological roles of pro-inflammatory cytokines such as TNFα, interleukin (IL)-1β, and IL-6 have been demonstrated. Therapeutic inhibitors of these targets, such as etanercept, a p75-TNFR immunoglobulin Fc fusion protein, infliximab, a TNF specific monoclonal antibody, and anakinra, an IL-1R antagonist, represent major treatment advances in this disease (reviewed in [1]). Nevertheless, a therapeutic response and efficacy are not always achieved and may be of limited duration. There is thus still a major need to understand pathways which sustain chronic inflammation in these diseases with the hope that treatment can be improved.

Nicotinamide adenine dinucleotide (NAD) is an important coenzyme found in all cells that plays key roles as carrier of electrons in the redox reaction, but also as cofactor for NAD-consuming enzymes. Evidence suggests that TNFα and other inflammatory stimuli affect NAD metabolism. For example, endotoxin, the potent stimulus of innate immunity, induces a dramatic increase in the expression of NAMPT, a crucial enzyme involved in the salvage pathway of NAD, recycling NAD from nicotinamide[2]–[4]. NAMPT was originally called pre-B-cell colony-enhancing factor (PBEF), a putative cytokine involved in B-cell development[5], and was later suggested to act as an adipokine secreted by visceral fat called visfatin[6]. The expression of NAMPT is upregulated during activation of immune cells such as monocytes, macrophages, dendritic cells, T cells, and B cells[4], [7]–[9], as well as in amniotic epithelial cells upon stimulation with lipopolysaccharide (LPS), TNFα, IL-1β, or IL-6[10]. Moreover, it was suggested that NAMPT has potential implications in the pathogenesis of acute lung injury[11], Crohn's disease (CD), ulcerative colitis (UC), and RA. Indeed, its expression is increased in colonic biopsy specimens of patients with CD and UC compared to healthy controls[12]. In RA, expression of NAMPT is upregulated in the inflamed synovial tissue of mice with antigen-induced arthritis, and in plasma and synovial fluid from RA patients[13]–[15]. However, the exact pathophysiological significance of this upregulation is still unknown. Finally, it has also been shown that this enzyme, found in an extracellular form, has pro-inflammatory as well as immunomodulating properties. In particular, recombinant NAMPT activated human leukocytes and synoviocytes and induced pro-inflammatory cytokines *in vitro,* and IL-6 upon injection in mice[12], [15].

APO866 (also known as FK866 and WK175) has been identified as a specific competitive low molecular weight inhibitor of NAMPT enzymatic function. The crystal structures of NAMPT, alone and in complex with the reaction product nicotinamide mononucleotide (NMN) or the inhibitor APO866, have been recently published[16], [17]. The structures showed that APO866 is bound in a tunnel at the interface of the NAMPT dimer, and competes directly with the nicotinamide substrate. Using tumor cell lines, it was found that APO866 inhibited NAMPT catalyzing the transformation of nicotinamide into NAD, but not a closely related enzyme transforming nicotinic acid into NAD. APO866 was thus found to deplete intracellular NAD content, resulting in apoptotic cell death in many cancer cell lines without any DNA damaging effect[18]–[20]. These data suggested the use of APO866 for treatment of diseases involving deregulated apoptosis, such as cancer. Here, we took advantage of the availability of this specific inhibitor to further explore the involvement of NAMPT enzymatic function in inflammatory arthritis.

## Results

### Expression of NAMPT is up-regulated in collagen-induced arthritis

**Figure 1:**
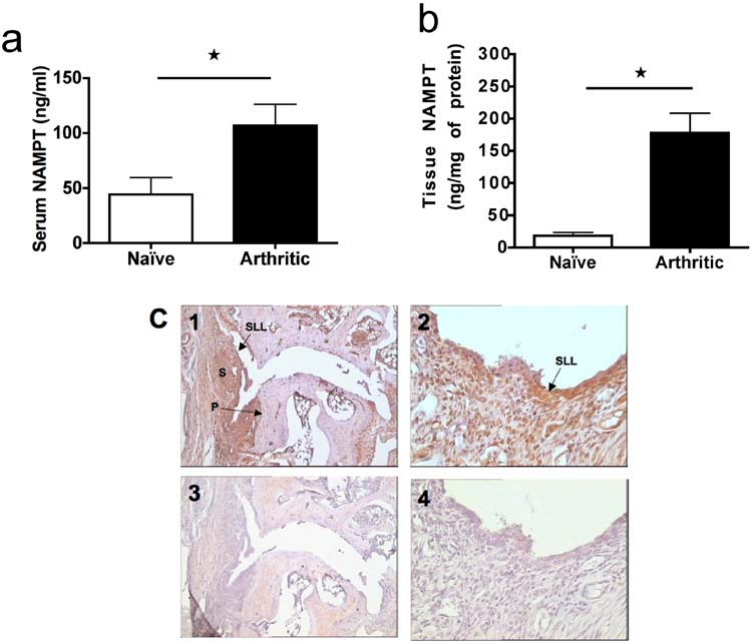
Induction of NAMPT expression in collagen-induced arthritis. Sera (a) and tissue extracts of paws (b) from CIA at day 14 (n = 8) and from non-arthritic, non-immunized, naïve (n = 7) mice were prepared and analyzed by NAMPT ELISA. *P<0.05 arthritic versus naïve in panel a and b. (c) NAMPT immunohistochemistry was performed on paw joints, using a specific rat anti-mouse NAMPT antibody (panels 1 and 2). Staining specificity was confirmed using an irrelevant isotype-matched antibody as primary antibody (panels 3 and 4). Synovial lining layer (SLL), synovial membrane (S) and pannus (P). Original magnifications: x100 for panels 1 and 3; x400 for panels 2 and 4.

We first examined expression of NAMPT during collagen-induced arthritis (CIA) in mice, a model that shares many histopathological features with RA in humans. During CIA, the level of NAMPT was significantly elevated in sera (*P* = 0.0288) and paw tissue extracts (*P* = 0.0025) from arthritic mice compared to non-arthritic naïve controls as measured by ELISA (Fig. 1a and b, respectively). These results were also supported by NAMPT immunohistochemistry. Indeed, we found massive staining of arthritic paw and knee joints from CIA (Fig. 1c), but markedly reduced staining in non-arthritic joints or joints from naïve mice (results not shown). In affected joints, NAMPT staining was prominent in synoviocytes of the synovial lining layer (SLL), sub-intimal synovium and pannus (P) and in some inflammatory cells (see arthritic paw, Fig 1c). Most of the blood vessels were also positive. In addition, some positive chondrocytes were observed in both normal and arthritic joints.

### NAMPT inhibition with APO866 reduces established collagen-induced arthritis

**Figure 2:**
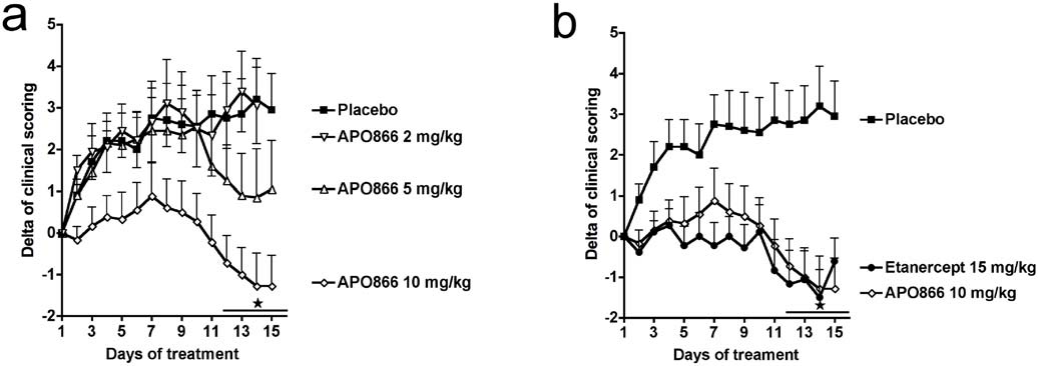
Effect of NAMPT inhibition with APO866 on established collagen-induced arthritis. (a) Dose-response effect of APO866: test mice were treated twice daily ip with APO866 2, 5, or 10 mg/kg (n = 10 in each group) during 15 days. Placebo mice received vehicle only (n = 10). (b) Severity of arthritis in CIA mice receiving APO866 10 mg/kg ip twice daily or etanercept 15 mg/kg every three days (n = 10 in each group) over 15 days. Mice groups were compared by two-way ANOVA. *P<0.05 APO866 or etanercept versus placebo in panel a and b.

**Figure 3:**
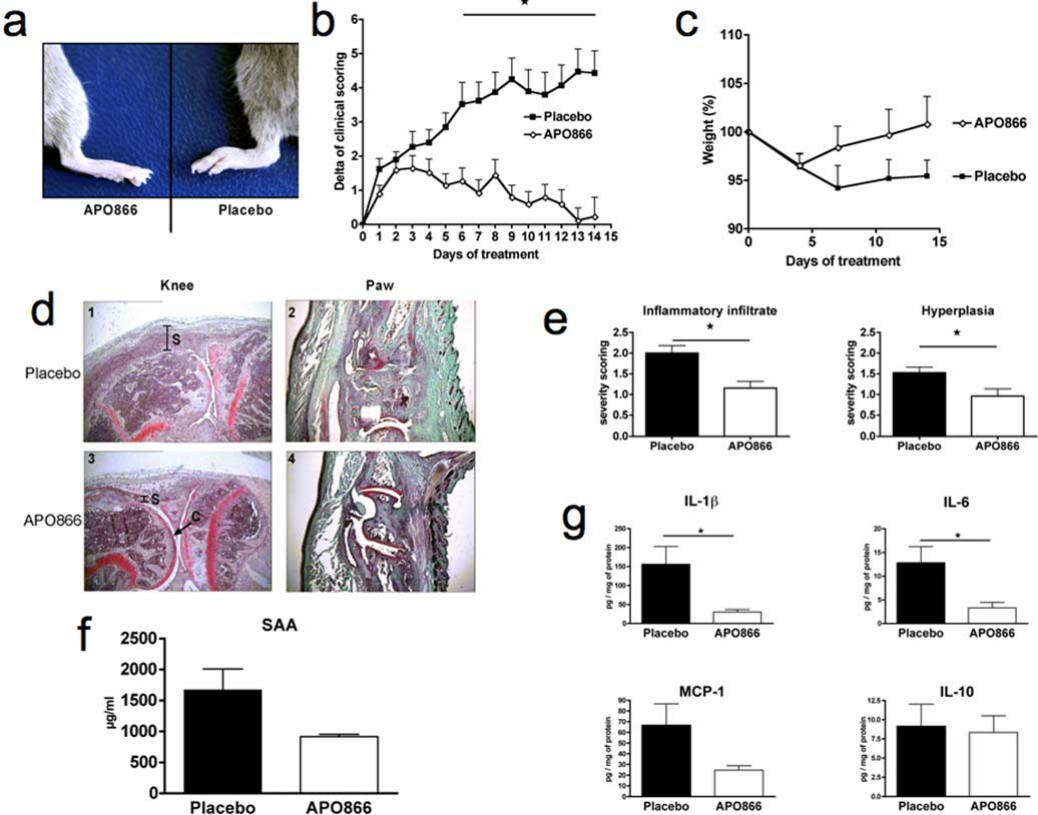
Clinical, histological and biochemical effects of NAMPT inhibition on established arthritis. Test mice (n = 20) were twice daily treated ip with 10 mg/kg of APO866 from the first day onward of appearance of clinical arthritis (clinical score >1) during 14 days. Placebo mice (n = 20) received vehicle only. (a) Representative photographs of paws of CIA mice APO866-treated or placebo-treated. Groups of animals were compared with respect to variation of their clinical scoring (b), and of their weight (c) by statistical analysis using the two-way ANOVA. (d) Histological features of arthritic joints: representative knee and paw histology from placebo and APO866-treated mice after 14 days of treatment. In the placebo group (pictures 1 and 2), the synovial membrane (noted S on picture) was significantly thicker than in treated animals (pictures 3 and 4). An effect on the articular cartilage (C) was also observed, with a decreased loss of Safranin-O staining in the treated group (compare panel 1 and 3 for knees and 2 and 4 for paws). Original magnification ×40. (e) A semi-quantitative histological evaluation was performed on the knee sections using a 4 points (0–3) scoring system to evaluate inflammatory infiltrate and synovial hyperplasia. (f) Circulating SAA levels: Sera from placebo- and APO866-treated CIA mice at day 14 (n = 8 and n = 7, respectively) were prepared and analyzed by SAA ELISA according to the manufacturer's instructions. (g) Cytokine levels in paw extracts: At the end of the experiment, IL-1β, IL-6, MCP-1, and IL-10 levels in paw extracts were determined as described in Methods. *P<0.05 APO866 versus placebo in panels b, e and g.

Having confirmed that NAMPT expression is increased in CIA, we next investigated if inhibition of NAMPT enzymatic function with APO866 could reduce established CIA. APO866 was administered from the day following the appearance of the first clinical symptoms of arthritis, and continued for 15 days. APO866 had a marked protective effect on CIA observed in 3 independent experiments, with a maximal therapeutic response when administered at 10 mg/kg (Fig. 2a). The beneficial effect was apparent within 10 days following the commencement of treatment and was evidenced by a reduced mean arthritic score (Fig. 2a), as well as by a lower mean number of affected paws (results not shown). We next wanted to compare the therapeutic effect of APO866 to the well-established effect of anti-TNFα treatment of CIA. APO866, at a dose of 10 mg/kg, was comparable to etanercept for inhibition of CIA (Fig. 2b). To gain more insight into the inhibitory mechanism of action of APO866 on CIA, we repeated the CIA curative experiment using the optimal dose of APO866 and analyzed more parameters. Paws from APO866-treated mice showed minimal signs of inflammation after 2 weeks of treatment whereas paws from placebo-treated mice were still inflamed (Fig. 3a), and this was also reflected in the clinical scoring (Fig. 3b). Additionally, these *in vivo* clinical observations were consistent with histology of knees and paws, where much less inflammation was observed in the APO866-treated group (Fig. 3d). Knee joints of placebo mice and mice treated with APO866 were assessed for inflammatory infiltrate and synovial hyperplasia. As shown in figure 3e, histological sections revealed a statistically significant decrease in inflammatory infiltrate (*P* = 0.0028) and hyperplasia (*P* = 0.0145) in mice treated with APO866 as compared to placebo-treated controls. Serum amyloid A protein (SAA) levels, which reflect the systemic inflammatory response, were decreased in APO866-treated mice (Fig. 3f), although this decrease did not reach significance (*P* = 0.0574), further suggesting the anti-inflammatory effect of APO866 administration. Amongst the potential molecular mechanisms involved in the amelioration of CIA by APO866 is the reduction of pro-inflammatory cytokines. Expression of different cytokines was investigated in paw tissue extracts at the end of the experiment. TNFα was below the level of detection of the assay. Locally produced IL-1β and IL-6 were significantly reduced (*P* = 0.0308 and *P* = 0.0396, respectively) in APO866-treated animals (Fig. 3g). MCP-1 was decreased, although this decrease was not statistically significant (*P* = 0.1103) (Fig. 3g), and finally a group composed of IL-10 (Fig. 3g), IFN-γ, CCL5, and IL-12p70 (not shown) remained unchanged by APO866 treatment (*P*>0.05).

We observed no signs of toxicity resulting from the treatment with APO866 since the weight of the mice was comparable between placebo- and APO866-treated groups (Fig. 3c). Indeed, APO866 was well tolerated, no premature death occurred in the treated group and the corresponding histopathology of liver, spleen, lung, gut, kidney, inguinal lymph nodes and brain in this group was no different from control animals. In addition, liver toxicity was also ruled out as similar low alanine aminotransferase levels were measured in APO866-treated versus control mice (data not shown). Importantly, no difference in the number of apoptotic cells between placebo and APO866-treated animals was observed *in situ* in affected paw tissues of arthritic mice (results not shown). Finally, hematological examination showed similarity between the treated and control mice (table 1).

**Table 1:** Haematological examination of vehicle and APO866-treated mice.

	PLACEBO		APO866	
	mean	SD	mean	SD
RBC (10^3^/mm^3^)	13.6	4.9	9.4	1.4
WBC (10^6^/mm^3^)	12.1	0.4	11.5	0.7
HGB (g/dl)	14.8	0.6	14.1	0.8
HCT (%)	55	2.4	52.8	2.6
PLT (10^3^/mm^3^)	1832.6	77	1688	181
				
%LYMPHO	54.6	7.6	60	7.4
%MONO	12.1	2.1	10.4	1.3
%NEUTRO	33.3	6.7	29.7	6.5

CIA was induced as described in Material and Methods. Test mice (n = 10) were twice daily treated ip with placebo or 10 mg/kg of APO866 from the first day onward of appearance of clinical arthritis (clinical score >1) during 14 days. At day 15, blood was collected in EDTA-coated tubes and cells counted with VetABC instrument.

To verify that APO866-treated mice generated an adequate immune response against type II collagen, total anti-collagen IgG levels were measured by ELISA at the end of the therapy (day 15). No significant difference was observed in anti-collagen IgG levels between control and APO866-treated mice (Vehicle-treated mice mice: 140+/−20.2 arbitrary units (n = 18), APO866-treated mice: 106.5+/−17.6 arbitrary units (n = 15)). Accordingly, cellular immune responses against type II collagen were similar between groups as assessed by inguinal lymph node T cell proliferation assays (results not shown). Collectively, these data show that the beneficial effects of APO866 on established CIA were neither due to toxicity nor to impaired immune response to collagen II, but suggest an impaired secretion of inflammatory cytokines.

### NAMPT inhibition reduces intracellular NAD concentration in inflammatory cells and circulating TNFα during endotoxemia

**Figure 4:**
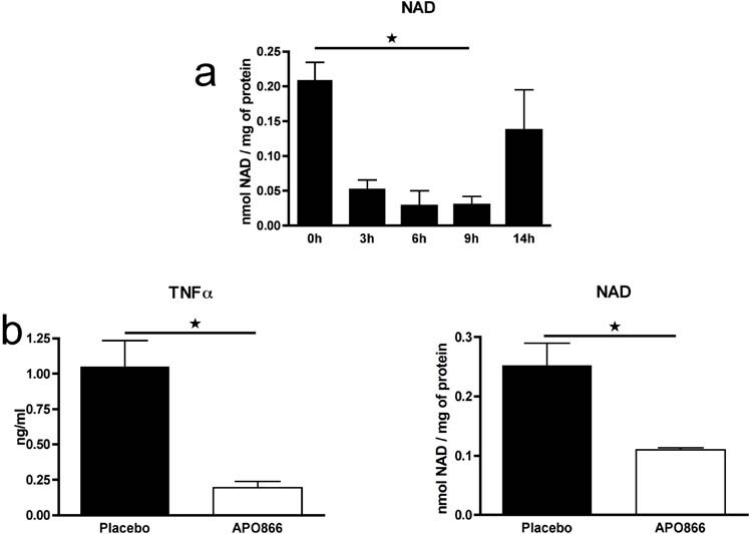
APO866 reduces intracellular NAD in PEC *in vivo* and inhibits TNFα production after LPS challenge. (a) Mice were treated with thioglycollate to elicit PEC, and then received 10 mg/kg APO866 by ip injection. PEC were obtained by lavage after different time points and intracellular NAD was determined. Data are mean+sem of 3 mice per group. (b) Mice were treated with thioglycollate to elicit PEC, and then received 10 mg/kg APO866 or placebo by ip injection 7 h before ip challenge with LPS. Serum TNFα at 90 min (mean+sem of 3 mice per group is shown. PEC were obtained by lavage and intracellular NAD was determined. Data are mean+sem of 3 mice per group. This panel is representative of at least 4 experiments performed. *P*<0.05 9 h versus 0 h in panel a, or APO866 versus placebo in panel b.

Next, we investigated the cellular target of NAMPT inhibition with APO866. We have not been able to measure intracellular NAD levels in inflammatory cells isolated from the paws of CIA mice due to the paucity of the cells and the difficulty to isolate them with high purity. To bypass these technical difficulties, we turned to a model of peritonitis where many inflammatory cells are recruited to the peritoneal cavity upon thioglycollate administration and are easily isolated with minimal manipulation. Thus, naïve mice were treated ip with thioglycollate to elicit inflammatory cells, and then APO866 was administered ip at 10 mg/kg. Peritoneal exudates inflammatory cells (PEC) were obtained by lavage at different time points after treatment, and intracellular NAD levels were determined using an enzymatic assay. Figure 4a shows that APO866 induced a significant time-dependent NAD depletion in PEC *in vivo* with a nadir at 9 h (*P* = 0.0248) and recovery around 14 h after injection (*P* = 0.3778). We also tested the ability of APO866 to reduce TNFα levels *in vivo* based on a classical model of experimental endotoxemia in mice. Naïve mice were treated ip with thioglycollate to elicit inflammatory cells, and then were treated ip with placebo or 10 mg/kg APO866 7 h before an ip injection of LPS. Mice were bled 90 min later for evaluation of serum TNFα levels. As shown in figure 4b, APO866 induced a significant decrease in circulating TNFα levels compared to placebo (*P* = 0.011). This decrease in TNFα secretion was accompanied by a significant decrease (*P* = 0.02) in intracellular NAD in PECs obtained from the same mice (Fig. 4b).

### Specific inhibition of NAMPT enzymatic function with APO866 reduces intracellular NAD concentration and pro-inflammatory cytokine production in mouse and human inflammatory cells

**Figure 5:**
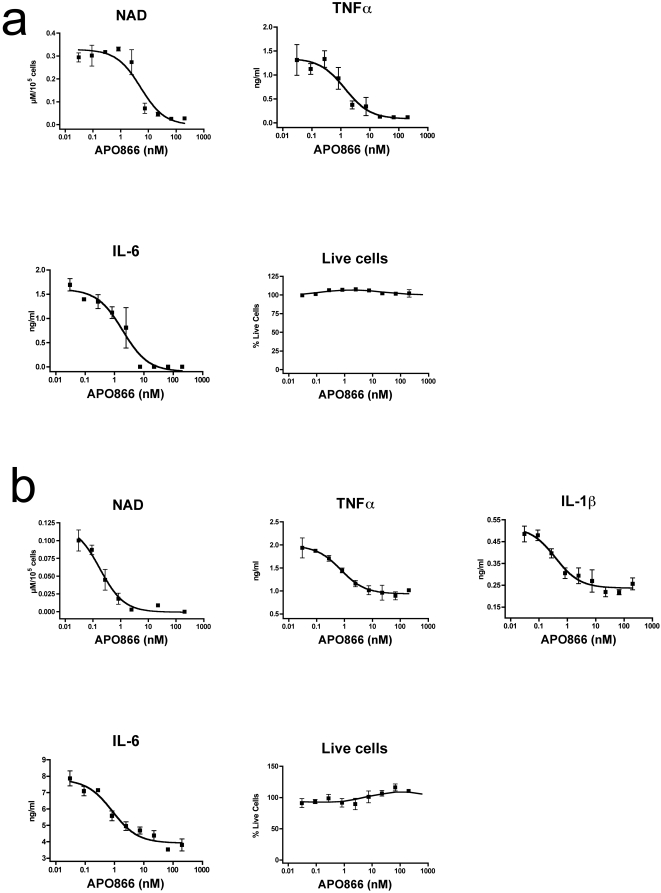
Inhibition of NAMPT enzymatic function with APO866 reduces intracellular NAD concentration and pro-inflammatory cytokine production in mouse and human inflammatory cells, without affecting viability. (a) Mouse PEC and (b) human monocytes were cultured for 4 h with increasing doses of APO866, and then stimulated overnight with SAC or LPS, respectively. At the end of the culture, the culture supernatants were tested for TNFα, IL-1β and IL-6 content by ELISA, cell viability was assessed using the Live/Dead kit, and intracellular NAD concentration was determined as described in Methods. Data are mean ± SEM of triplicates. This panel is representative of at least 3 experiments performed.

To gain further insight in the potential molecular mechanisms involved in the amelioration of inflammation in CIA by APO866, the effect of pharmacological inhibition of NAMPT with APO866 on highly purified inflammatory cells was determined *in vitro* in a closed system. To this end, thioglycollate-elicited mouse inflammatory peritoneal cells (PEC) were cultured for 4 h with increasing doses of APO866, and then stimulated overnight with Pansorbin (heat-killed, formalin fixed Staphylococcus aureus Cowan I cells (SAC)) to induce inflammatory cytokines secretion. Intracellular NAD level was measured using an enzymatic assay and culture supernatants were tested for TNFα, IL-1β and IL-6 content by ELISA. IL-1β was below the level of detection of the assay. In this system, APO866 reduced in a dose-dependent manner both intracellular NAD concentration and pro-inflammatory cytokines TNFα, and IL-6 (Fig. 5a). Similar results were obtained upon stimulation with LPS (data not shown). Importantly, inhibition of pro-inflammatory cytokine secretion was not due to cell death induction, as in the same culture conditions, APO866 did not affect cell survival, as assessed by intracellular esterase activity and DNA incorporation of ethidium bromide (Fig. 5a) or AnnexinV-propidium iodide staining, cell count, or trypan blue exclusion (data not shown).

**Figure 6:**
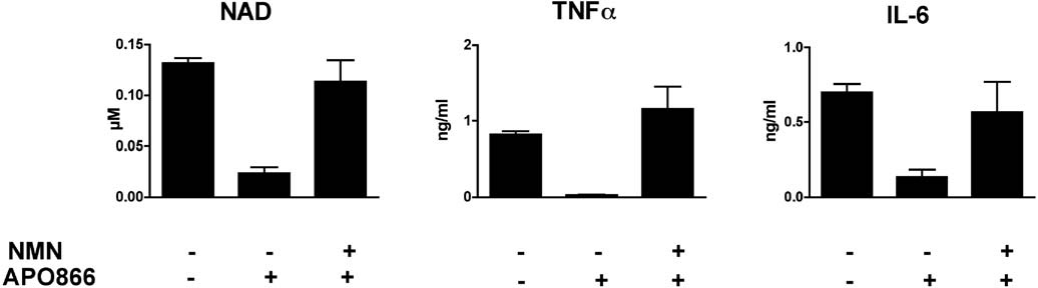
Exogenous nicotinamide mononucleotide, NAMPT-catalyzed reaction end product, reverts the inhibitory effects of APO866. Mouse PEC were incubated in the presence or absence of 200 nM APO866 and 10 mM nicotinamide mononucleotide (NMN). Cells were further stimulated with LPS and intracellular NAD levels and TNFα and IL-6 secretion were determined. Data are mean±SEM of triplicates.

Anti-inflammatory properties of APO866 were then tested on human cells. Human monocytes isolated from PBMC obtained from healthy volunteers were cultured for 4 h with increasing doses of APO866, and then stimulated overnight with LPS. As observed for mouse cells, APO866 inhibited NAD, TNFα, IL-1β and IL-6 in a dose-dependent fashion, while preserving cell viability (Fig. 5b). Similar results were obtained using other inflammatory human cells, such as total PBMCs, and human monocyte-derived dendritic cells, or upon stimulation with SAC (data not shown). Thus, inhibition of NAMPT enzymatic function with APO866 efficiently reduced both human and mouse cell pro-inflammatory cytokines secretion by a mechanism independent of cell death. To further emphasize the specificity of the APO866 for its target NAMPT, PECs were incubated for 4 h with or without APO866 and exogenous NMN, the product of the NAMPT-catalyzed reaction inhibited by APO866. The cells were then stimulated overnight with LPS to induce pro-inflammatory cytokines secretion. As shown in figure 6, addition of NMN restored both intracellular NAD levels and TNFα and IL-6 production in PEC even in the presence of APO866, further supporting the notion that NAMPT represents the only molecular target of APO866. Moreover, these results strongly support the contention that the link between visfatin/NAMPT and inflammation might be only related to its enzymatic activity as a NAD biosynthetic enzyme.

## Discussion

In the present article, we have identified anti-inflammatory properties of APO866 by inhibition of NAMPT enzymatic function. In CIA, NAMPT expression was increased and therapeutic administration of its inhibitor APO866 ameliorated disease severity, and no weight loss or other signs of toxicity were observed. The inhibition of CIA by APO866 at the dose used was as efficient as TNFα blockade by etanercept and was associated with decreased local IL-1β and IL-6 secretion. In the same arthritic paw tissue extracts, TNFα was below the detection limit but this does not preclude a role for TNFα in this model since TNFα could act early on in the disease process. Moreover, a single ip administration of APO866 at a dose of 10 mg/kg lowered intracellular NAD levels in inflammatory cells in a time-dependent fashion and circulating TNFα was reduced following an endotoxin challenge *in vivo. In vitro,* NAMPT inhibition was found to reduce TNFα, IL-1β, and IL-6 secretion upon stimulation of inflammatory cells with bacterial agents. Moreover, the inhibition of pro-inflammatory cytokines correlated with a decreased intracellular NAD concentration in inflammatory cells, while the viability of the cells was not affected. The effect of APO866 was reversed by supplementation with NMN, the NAMPT-catalyzed reaction product, further confirming data on the specificity of APO866 for NAMPT obtained in previous studies[16]–[20]. Our study suggests that inhibition of NAMPT enzymatic function and reduction of intracellular NAD content by low doses of APO866 might be an effective treatment of certain autoimmune or autoinflammatory disorders, in particular those where cytokines such as TNFα, IL-1β, and IL-6 have been shown to play a major role in the initiation and maintenance of the disease.

Inhibition of TNFα, IL-1β, and IL-6 production is beneficial in several inflammatory diseases including arthritis and numerous efforts have been devoted in the design of novel therapies aimed at blocking the production and/or the biological effects of these important pro-inflammatory cytokines[21]. The exact molecular mechanism linking NAMPT inhibition by APO866 and blockade of cytokine production is still a matter of speculation, but several avenues are open for discussion. Among other functions, NAD is a co-factor for NAD-consuming enzymes with multiple roles in cellular regulation[22], such as mono-ADP-ribosyltransferases (mARTs), poly(ADP-ribose) polymerases (PARPs), and sirtuins [23], [24]. A member of the PARP family, PARP-1, has been proposed to act as a co-activator of NF-κB during the pathogenesis of inflammatory disorders, including RA[25]. By decreasing intracellular NAD levels, APO866 might indirectly affect PARP-1 function and alter the expression of pro-inflammatory cytokines. Alternatively, by inhibiting NAMPT, and preventing the transformation of nicotinamide into NAD, APO866 could also indirectly alter inflammation by inducing the accumulation of nicotinamide, which is a known anti-inflammatory agent. Indeed, nicotinamide protects from the toxic effects of staphylococcal enterotoxin B[26], and inhibits LPS-induced TNFα *in vivo* in mice[27]. Finally, the inhibitory effect of APO866 on TNFα could be accounted for by its impact on sirtuins as it was shown that NAMPT could regulate the activity of NAD-dependent Sirt1 in mammalian cells[28].

It has been recently reported that NAMPT is present in an extracellular secreted form and can regulate activation of human leukocytes and synoviocytes by increasing surface expression of costimulatory molecules and by inducing IL-1β, IL-6, and TNFα production through a putative membrane receptor[12], [15]. In addition, NAMPT was shown to be produced by visceral fat as a secreted adipokine called visfatin that exhibited insulin mimetic functions through binding to the insulin receptor[6]. At present, the exact mechanism of action of this extracellular protein on the activation of immune cells or in insulin regulation is a matter of controversy. Indeed, the article describing the insulin mimetic function of visfatin actin through the insulin receptor has been retracted from Science[29], and Revollo *et al*. have very recently shown that NAMPT/PBEF/visfatin functions as an intra- and extracellular NAD biosynthetic enzyme and these authors were unable to reproduce the insulin-mimetic activity of visfatin[30]. In this context, we have been unable to reproduce the reported results supporting the notion that recombinant visfatin may act as an extracellular pro-inflammatory cytokine through binding to a putative receptor on human PBMCs, even at doses above the physiologocal levels (results not shown). In addition, our *in vitro* results with inflammatory cells show that the inhibition of TNFα and IL-6 by the specific inhibitor of NAMPT APO866 was accompanied by a decrease in intracellular NAD and both intracellular NAD and TNFα and IL-6 secretion were reversed by addition of NMN even in the presence of APO866, further supporting the notion that the link between visfatin/NAMPT and inflammation might be only related to its enzymatic activity as a NAD biosynthetic enzyme.

The question whether NAMPT, a protein lacking a typical signal sequence for secretion, is actively secreted or found in extracellular compartments because of passive diffusion upon cell death remains open. Indeed, chronic inflammation, especially in RA, is associated with tissue damage and remodeling[31]. In this context, cell lysis could be responsible for the release of NAMPT in the extracellular compartment thus accounting for the increased NAMPT concentration found in the serum and tissues of RA patients and in mouse arthritis models ([13]–[15] and our results). Of note, two groups recently reported that NAMPT is positively secreted through a non-classical secretory pathway in adipocytes and transfected CHO cells[32], [33].

It is not unknown for anti-cancer agents to be used as anti-inflammation drugs, histone deacetylase inhibitors and methotrexate being two such examples[34], [35]. APO866 is currently in several Phase II clinical trials to evaluate its efficacy in controlling cancer growth. *In vitro*, incubation of tumor cells results in the depletion of intracellular NAD, and activation of the apoptotic cascade with release of cytochrome *c*, and activation of caspase 3, but without any DNA damaging effect or alteration in p53 expression[18]–[20]. Furthermore, apoptosis is dramatically enhanced when APO866 is combined with agents inducing genotoxic stress ([36] and our own unpublished results), suggesting that the apoptosis observed in tumor cells with APO866 is related to intrinsic DNA instability and DNA repair activities found in transformed cells. On the contrary, the anti-inflammatory effects of NAMPT inhibition through APO866 were not associated with increased cell death of inflammatory cells *in vitro* and no difference in the content of apoptotic cells between placebo and APO866-treated animals was observed *in situ* in affected tissues of arthritic mice (this article). Moreover, higher doses of APO866 are required for reduction of tumor burden in mice compared to those altering inflammation (our own unpublished observations), suggesting different effector mechanisms for apoptosis of tumor cells and inhibition of pro-inflammatory cytokine secretion in inflammatory cells.

Hypoxia can be a leading cause of angiogenesis. Recent data provide evidence that NAMPT is up-regulated by hypoxia through hypoxia-inducible factor 1[37], [38] and demonstrate that NAMPT promotes *in vitro* and *in vivo* angiogenesis via activation of mitogen-activated protein kinase ERK-dependent pathway[39], thus suggesting that NAMPT/visfatin might play important roles in various angiogenesis-related disorders. In this context, the abnormally elevated NAMPT levels reported in human and experimental arthritis ([13], [14], and our results on CIA) could well result from the hypoxic conditions in the rheumatoid synovial microenvironment [40], [41] further contributing to the angiogenic process found in RA[42]. Indeed, anti-angiogenic therapies have been successfully tested in experimental models of arthritis, such as CIA[43]. It is interesting to note that APO866 has been previously reported to have anti-angiogenic activity *in vivo* in a murine model of renal cell carcinoma at the same dose efficient in our CIA experiments[44]. The inhibitory effect of APO866 in CIA might thus be accounted for, at least in part, by its anti-antiangiogenic activity.

In conclusion, the present paper establishes a new functional link between NAD metabolism and inflammation, and suggests a potential important role for NAD-dependent enzymes in the regulation of pro-inflammatory cytokine production, including TNF, IL-1β, and IL-6. Our data identify a new molecular pathway that can lead to the development of novel therapeutics for the treatment of inflammatory diseases. NAMPT has been described to be a relevant clinical biomarker in a series of inflammatory-related disorders and now our results suggest that NAMPT is an important actor in the pathology of these diseases. These findings open the possibility to test quite rapidly the clinical efficacy of NAMPT inhibition in inflammatory diseases in man, raising new hopes for the development of effective treatments for debilitating diseases.

## Methods

### Mice

Male DBA/1 and female or male C57BL/6 mice between 8–12 weeks of age were obtained from Harlan (Horst, The Netherlands). Animals were housed under conventional conditions, water and standard laboratory chow were provided *ad libitum*. All animal experiments were approved by a local ethics committee (Service Vétérinaire Cantonal, Lausanne, Switzerland).

### *In vitro* test of APO866 function

Human blood was obtained from consenting donors and peripheral blood mononuclear cells (PBMC) were isolated using centrifugation over Ficoll-Paque PLUS cushions (Amersham Biosciences, Uppsala, Sweden). After washing, cells were aliquoted at 10 million per vial and frozen in 90 % fetal calf serum (FCS) 10% DMSO (vol/vol). For *in vitro* stimulations, cells were thawed, washed, and monocytes were isolated by negative depletion using the Monocyte Isolation kit II from Miltenyi Biotec (Bergisch Gladbach, Germany) according to manufacturer's instructions. Purity was more than 90% as shown by flow cytometry staining with CD14. Cells were resuspended in RPMI 1640 Glutamax (Invitrogen) containing 10% FCS and 1% penicillin-streptomycin, plated at 1×10^5^ /well (for viability and NAD measurement) or 2×10^4^ /well (for cytokine measurement) of a 96-well flat bottom plate, and incubated for 4 h at 37°C with graded concentrations of (E)-N-[4-(1-benzoylpiperidin-4-yl) butyl]-3-(pyridine-3-yl)-acrylamide (APO866, synthesized and provided by Astellas Pharma GmbH, Munich, Germany), after which the cells were stimulated with 100 ng/ml lipopolysaccharide (LPS from *Escherichia coli* 0111:B4, Sigma, St. Louis, MO, USA) in a final volume of 220 μl per well. After overnight culture at 37°C, the supernatants were removed, diluted to avoid saturation of the ELISA, and assayed for cytokine content using DuoSet kits from R&D Systems Europe (Abingdon, UK). Cell viability was measured using the Live/Dead viability/cytotoxicity kit from Molecular Probes (Eugene, OR, USA). Intracellular NAD content was determined as described[45].

Peritoneal exudate cells (PEC) were used for studies with mouse cells. Briefly, naive mice were injected ip with 1 ml 4% thioglycollate (BBL thioglycollate medium brewer modified, BD, Sparks, MD, USA). 5 days later, PEC were obtained by lavage of the peritoneal cavity with 10 ml cold PBS. After washing, cells were aliquoted at 10 million per vial and frozen in 90% FCS 10% DMSO (vol/vol). Culture conditions and stimulations were carried out as described above for human monocytes, except that the cells were stimulated with 5 μg/ml Pansorbin (heat-killed, formalin-fixed *Staphylococcus aureus* cells, SAC, Calbiochem, Nottingham, UK)) instead of LPS. To assess the specificity of the drug, PEC were incubated for 4 h in the presence or absence of APO866 (200 nM) and nicotinamide mononucleotide 10 mM (Sigma). Cells were then stimulated with 100 ng/ml LPS overnight. Cytokines and NAD content were measured as described above.

### Kinetics of NAD depletion in PEC after APO866 treatment *in vivo*

Naïve C57BL/6 mice were injected ip with 1 ml 4% thioglycollate to elicit PEC. 5 days later, mice were treated ip with 10 mg/kg APO866, and PEC were obtained by lavage of the peritoneal cavity with 10 ml cold PBS at different time points thereafter. Intracellular NAD content was determined, and results were normalized for total protein content using the Micro BCA kit according to manufacturer's instructions (Pierce, Rockford, IL, USA).

### LPS induction of serum cytokines *in vivo*

Naïve C57BL/6 mice were injected ip with 1 ml 4% thioglycollate to elicit PEC. 5 days later, mice were injected ip with placebo or 10 mg/kg APO866, and, after 7 h, were injected ip with 1 μg LPS. 90 min later, blood was obtained from anesthetized animals. Diluted sera were assayed for TNFα levels using a DuoSet ELISA (R&D Systems). Mice were subsequently killed by CO_2_ exposure, and PEC were obtained by lavage of the peritoneal cavity with 10 ml cold PBS. Intracellular NAD content was determined, and results were normalized for total protein content using the Micro BCA kit as described above.

### Induction of collagen-induced arthritis

Native chicken or bovine type II collagen (CII) was purchased from Morwell Diagnostics (Zumikon, Switzerland) and was dissolved at 2 mg/ml in 0.1 M acetic acid. Male DBA/1 mice were immunized with 100 μg of native CII, emulsified in incomplete Freund's adjuvant containing 5 mg/ml mycobacterium tuberculosis, by intradermal injection at the base of tail. On day 21, a booster injection of 100 μg collagen in incomplete Freund's adjuvant was given at the base of the tail. All immunization reagents were purchased from Difco (Basel, Switzerland). From day 21 after the first immunization onward, mice were examined daily for the onset of clinical arthritis. The severity of arthritis was scored on a 3-point scale, where 0 = normal appearance, 1 = mild swelling and/or erythema, 2 = pronounced swelling and erythema, and 3 = joint rigidity. Each limb was graded, resulting in a maximal clinical score of 12 per animal. A stock solution of APO866 at 10 mg/ml in PG 60% in water was diluted 5-fold in 0.9% NaCl and administered ip at 2, 5, and 10 mg/kg twice daily every 12 hours. Control mice received the same amount of vehicle (PG 12% in 0.9% NaCl). The treatment was administered for a total of 14–15 days from the day following appearance of the first clinical symptoms of arthritis (clinical scoring ≥1), with no differences in scoring between experimental groups at the onset of treatment. To eliminate any bias in the experiment, clinical scoring of the mice was done by an observer unaware of the identity of the treatment (placebo versus APO866 at 2, 5, or 10 mg/kg twice daily by the ip route). Etanercept (Enbrel, Wyeth, Zoug, Switzerland), used as a positive control, was injected ip at 15 mg/kg every three days starting from the day following appearance of clinical arthritis. All mice were sacrificed 14–15 days after CIA became clinically detectable.

### Histological analysis

Paws and knees were dissected and fixed in 10% buffered formalin for 7 days. Fixed tissues were decalcified for 3 weeks in 15% EDTA, dehydrated and embedded in paraffin. Sagittal sections (8 μm) of the whole knee joint were stained with safranin-O and counterstained with fast green/iron hematoxylin. Histological sections were graded independently by two observers unaware of animal treatment using an established scoring system for synovial hyperplasia (from 0: no hyperplasia, to 3: most severe hyperplasia) and inflammatory cells in synovial fluid (from 0: no inflammation, to 3: severe inflamed joint fluid).

### Haematological examination

Blood was collected at the end of the experiment by tail vein bleeding in EDTA-coated tubes and blood cells were enumerated by VetABC instrument (Medical Solution GMBH, Steinhausen, Switzerland).

### Measurement of serum amyloid A (SAA), and CII-reactive antibodies

Blood was collected at the end of the experiment by cardiac puncture. Serum levels of SAA were determined using a direct ELISA according to manufacturer's instructions (Biosource Europe, Nivelles, Belgium). Serum levels of total anti-mouse CII antibodies were determined using a commercial ELISA (Chondrex, Morwell Diagnostics).

### Tissue protein extracts preparation

At the end of the experiment, mice were killed and the left hind paw was frozen. Paws were cut into little pieces and homogenized in PBS containing Roche complete protease inhibitors (Roche, Basel, Switzerland) using an Ultratorrax T8 homogenizer (IKA, Staufen, Germany). The homogenates were centrifuged at 15000 g for 15 min at 4°C, and the supernatants stored at −20° C. Cytokine content was evaluated using the BD mouse inflammation cytometric bead array (CBA) kit (BD, Basel, Switzerland) or using a DuoSet ELISA kit for determination of IL-1β (R&D systems). Results were normalized for total protein content using the Micro BCA kit according to manufacturer's instructions (Pierce, Rockford, IL, USA).

### NAMPT expression

NAMPT concentration was determined in murine sera and tissue extracts using a mouse visfatin/PBEF ELISA kit (Circulex, LabForce, Nunningen, Switzerland)

NAMPT localization was studied by immunohistochemistry (IHC) using a rat monoclonal antibody against murine NAMPT[4] on paraffin-embedded sections of paws. Briefly, deparaffinized and rehydrated sections were incubated for 30 min at room temperature with 5% BSA and 20% normal rabbit serum. Endogenous peroxidase activity was blocked with 3% H_2_O_2_ for 10 min. Slides were then overlaid with the primary antibody at 5 μg/ml for 1 h at room temperature, followed by a biotinylated anti-rat mAb. Bound antibody was visualized using the avidin-biotin-peroxidase complex (Vectastain Elite ABC kit, Vector Laboratories, Burlingame, CA, USA). The color was developed by 3,3′-diaminobenzidine (Sigma Chemical Company) containing 0.01% H_2_O_2_ and slides were counterstained with Papanicolaou. Staining specificity was confirmed using an isotype-matched antibody as primary antibody.

### Statistical analysis

For values with non-Gaussian distribution, the significance of differences was calculated using the Mann-Withney *U* test for unpaired variables or the Wilcoxon test. For normally distributed variables, the significance of differences was analyzed using the Prism software (GraphPad software, Inc., Version 4) using a two-tailed Student *t* test for single time point measure or a two-way ANOVA for time course assay. All values were expressed as mean+/−SEM. A difference between experimental groups was considered significant when the *P* value was <0.05.
